# The *Pseudomonas aeruginosa* AlgZR two-component system coordinates multiple phenotypes

**DOI:** 10.3389/fcimb.2014.00082

**Published:** 2014-06-20

**Authors:** Yuta Okkotsu, Alexander S. Little, Michael J. Schurr

**Affiliations:** Department of Microbiology, University of Colorado School of MedicineAurora, CO, USA

**Keywords:** *Pseudomonas aeruginosa*, two-component regulation, AlgZR, alginate, twitching motility, LytTR family

## Abstract

*Pseudomonas aeruginosa* is an opportunistic pathogen that causes a multitude of infections. These infections can occur at almost any site in the body and are usually associated with a breach of the innate immune system. One of the prominent sites where *P. aeruginosa* causes chronic infections is within the lungs of cystic fibrosis patients. *P. aeruginosa* uses two-component systems that sense environmental changes to differentially express virulence factors that cause both acute and chronic infections. The *P. aeruginosa* AlgZR two component system is one of its global regulatory systems that affects the organism's fitness in a broad manner. This two-component system is absolutely required for two *P. aeruginosa* phenotypes: twitching motility and alginate production, indicating its importance in both chronic and acute infections. Additionally, global transcriptome analyses indicate that it regulates the expression of many different genes, including those associated with quorum sensing, type IV pili, type III secretion system, anaerobic metabolism, cyanide and rhamnolipid production. This review examines the complex AlgZR regulatory network, what is known about the structure and function of each protein, and how it relates to the organism's ability to cause infections.

## Significance of *P. aeruginosa*

*Pseudomonas aeruginosa* is a ubiquitous, metabolically versatile, environmental organism with the ability to cause opportunistic infections in humans. Its >6 Mbp genome contains at least 5500 open reading frames (ORFs), encoding hundreds of virulence determinants (Wolfgang et al., 2003a,b; Winsor et al., 2011). *P. aeruginosa* is the predominant bacterial pathogen in cystic fibrosis (CF) patients, where colonization by *P. aeruginosa* in the CF lung is linked to a worsening disease prognosis (Henry et al., 1982, 1992; Nixon et al., 2001). It is also a significant cause of hospital-acquired infections (Almirante et al., 2012; Orsi et al., 2012; Horcajada et al., 2013; Khawaja et al., 2013; Simonetti et al., 2013), particularly in burn-wounds (Regules et al., 2008; Belba et al., 2013), and immunocompromised individuals (Gomes et al., 2011; Papagheorghe, 2012; Sousa et al., 2013). *P. aeruginosa* is able to form multiple types of biofilms, which allow them to persistently colonize a variety of surfaces, thereby making their eradication extremely challenging.

## *P. aeruginosa* in the CF lung and the isolation of mucoid isolates

**Figure 1:**
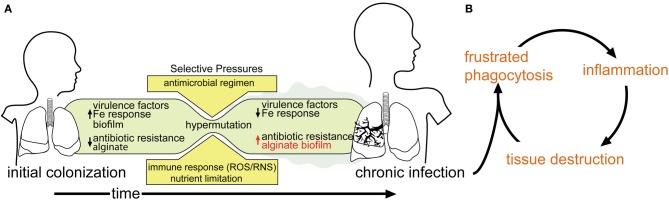
**Model of *P. aeruginosa* infection within a CF patient**. **(A)** As infection proceeds to a chronic state, phenotypes associated with chronic infection (e.g., mucoidy) predominate, while acute virulence factors are selected against. **(B)** The inability of the host-immune response to clear chronically established infection leads to a debilitating cycle of inflammation and tissue destruction.

Respiratory *P. aeruginosa* infection in CF patients occurs intermittently between 6 months and 13 years of life, usually from an environmental reservoir (Johansen and Hoiby, 1992; Armstrong et al., 1995; Burns et al., 2001; Li et al., 2005; Ranganathan et al., 2013). Bacteria in the CF lung are constantly under stress due to: (i) the notably dehydrated airway mucus associated with this disease; (ii) bombardment from administered antibiotics; (iii) attack from host antibacterial compounds; and (iv) oxidative stress related to immune system assault (Hartl et al., 2012). Over time, bacteria in the CF lung, including *P. aeruginosa*, may adapt to these and other stresses and establish a chronic infection (Figure 1A). The hallmark phenotype of a chronic *P. aeruginosa* infection is overproduction of an exopolysaccharide matrix (Doggett et al., 1966). It is comprised of alginic acid (alginate), a polysaccharide consisting of a (1–4) linked β-D-mannuronate and α-L-guluronate copolymer. Strains over-expressing alginate exhibit a mucoid phenotype (Linker and Jones, 1964; Doggett, 1969; Evans and Linker, 1973).

Colonization by mucoid *P. aeruginosa* is associated with a significant decline in pulmonary function and disease outcome (Ballmann et al., 1998; Parad et al., 1999; Li et al., 2005). Alginate serves as a protective barrier for *P. aeruginosa* that resists opsonization and phagocytosis (Schwarzmann and Boring, 1971; Stiver et al., 1988; Pier et al., 1990; Leid et al., 2005), increases its resistance to some antibiotics (e.g., tobramycin) (Hentzer et al., 2001), as well as desiccation (Chang et al., 2007). Macrophages and neutrophils that are recruited through inflammatory chemotaxis are unable to clear the *P. aeruginosa* infection, and this “frustrated phagocytosis” leads to extensive auto-inflammatory lung damage (Pederson et al., 1992) (Figure 1B). Therefore, elucidating the molecular and biochemical mechanisms of alginate overproduction has been an area of active study for the last 40 years.

**Figure 2:**
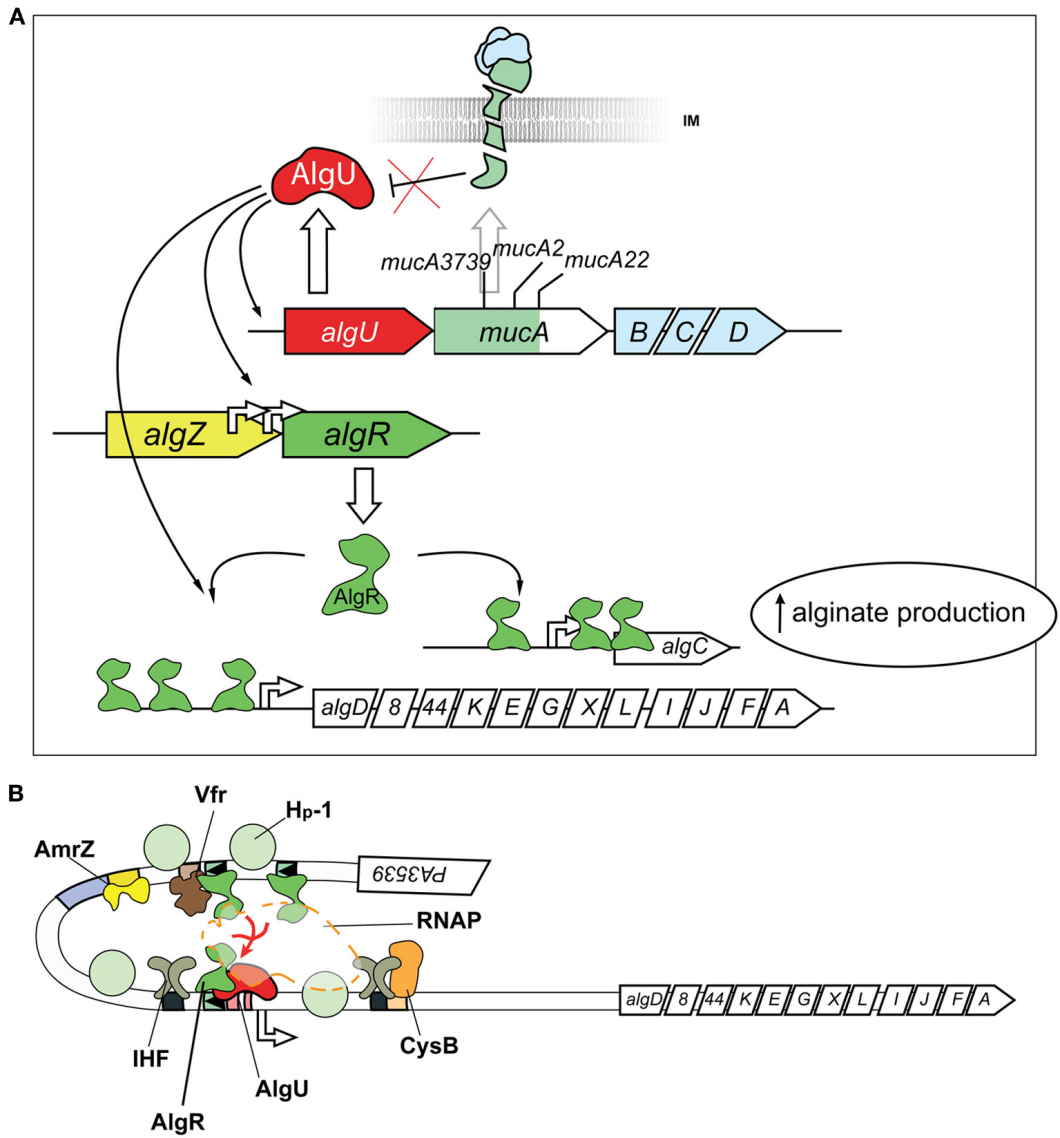
**Post-Translational and Transcriptional controls for alginate production**. **(A)** Post-translational activation of the alternative sigma factor AlgU (AlgT) is required for alginate production. Release of AlgU (AlgT) by MucA (depicted as a broken green transmembrane comma) through mutation (*mucA3739, mucA2, mucA22*) or degradation by proteases (see text) activates expression of itself, *algR*, and *algD*. AlgU (AlgT) and AlgR coordinate activation of the *algD* promoter along with other transcriptional activators (see **B**). Gene names are labeled within arrowed boxes, and pointed end of boxes indicate open coding direction. **(B)** Transcriptional model for *algD* promoter activation. At least seven different transcriptional regulators activate the *algD* promoter, causing a DNA looping of the promoter region. The following proteins bind and regulate *algD* transcription: (i) AlgR; (ii) AlgU (AlgT, α^22^), an alternative sigma factor and RpoE ortholog (iii) AmrZ, a positive regulator of the ribbon-helix-ribbon family of proteins (Baynham and Wozniak, 1996; Baynham et al., 1999, 2006; Pryor et al., 2012); (iv) Integration Host Factor (IHF), a histone-like protein (Mohr et al., 1992; Toussaint et al., 1993); (v) AlgP (AlgR3, Hp-1), C-terminus histone-like protein (Deretic and Konyecsni, 1990; Deretic et al., 1992a); (vi) the activator CysB, a LysR-like transcriptional regulator (Delic-Attree et al., 1997); and (vii) Vfr.

One of the mechanisms for mucoid conversion in *P. aeruginosa* is associated with an extracytoplasmic function (ECF) sigma/anti-sigma factor system, encoded by the *algU/TmucABCD* operon (PA0762–0766) (Figure 2A). The *algU/TmucABCD* system is analogous to the well-characterized ECF sigma factor class (σ^E^) envelope stress response system in *Escherchia coli* (Rowley et al., 2006), which responds to environmental stressors, including reactive oxygen species, antimicrobial peptides, stationary phase, and carbon starvation that contribute to cell wall perturbations (Yu et al., 1995; Schurr et al., 1996; Yu et al., 1996; Barchinger and Ades, 2013).

In non-mucoid *P. aeruginosa*, the MucA anti-sigma factor, encoded by the *mucA* gene, sequesters the stress-responsive AlgU/T sigma factor to the inner-membrane (Figure 2A) (Hershberger et al., 1995; Schurr et al., 1995, 1996; Xie et al., 1996). Sequestration of the sigma factor to the membrane prevents the transcriptionally competent AlgU/T- RNA polymerase (RNAP) complex from transcribing AlgU regulated genes, including the expression of alginate biosynthetic enzymes encoded on the PA3540–3551 operon, the *algR* (PA5261) gene (Rowen and Deretic, 2000) and many others (see below) (Firoved et al., 2002; Firoved and Deretic, 2003; Tart et al., 2006; Wood and Ohman, 2009).

Alginate overproduction is regulated at two general levels: at the post-translational level as a response to environmental stress (e.g., iron, phosphate, or carbon limitation), and at the genetic level from mutations in the chromosome. At the post-translational level, alginate biosynthesis is activated by the regulated proteolytic degradation of MucA. Several excellent reviews describing the details of post-translational regulation and biosynthesis of alginate overproduction are currently available (Govan and Deretic, 1996; Franklin et al., 2011; Damron and Goldberg, 2012; Okkotsu et al., 2013a; Wiens et al., 2014). At the genetic level, alginate overproduction occurs as a function of mutations in the chromosome. The *P. aeruginosa* mismatch repair (MMR) system and the 7,8-dihydro-8-oxo-deoxyguanine repair (GO) system are two levels of genomic repair that are commonly impaired during chronic infection. Mutations in these systems result in highly mutable strains (Oliver et al., 2000; Hogardt et al., 2007; Mena et al., 2008). There is an increase in hypermutable *P. aeruginosa* variants during chronic respiratory infections (Hogardt et al., 2007) thereby enabling a multitude of phenotypic variants to arise from an isogenic progenitor (Oliver et al., 2000). Another mechanism by which mutations potentially arise is through an epistatic effect of DinB (pol IV), an error-prone DNA polymerase, and MutS when the organism is exposed to hydrogen peroxide (Sanders et al., 2006, 2011). During chronic CF pulmonary infections, mutations most commonly arise in the *mucA, mucB*, and *mucD* genes resulting in premature stop-codons, frame-shifts, and missense mutations. The most common *mucA* mutation found in *P. aeruginosa* isolates from CF patients is a deletion in a homopolymeric guanine tract at △G430 of *mucA* (Martin et al., 1993b; Boucher et al., 1997; Anthony et al., 2002; Bragonzi et al., 2006; Ciofu et al., 2008). The consequence of these mutations is that MucA, MucB, or MucD either become non-functional, have reduced function or exhibit increased susceptibility to cellular proteases. These defects ultimately result in the release of AlgU/T to the cytoplasm and activation of the AlgU/T regulon, including its own expression, genes for alginate production, and production of lipoproteins (Firoved et al., 2002; Firoved and Deretic, 2003; Firoved et al., 2004; Wood et al., 2006).

## Significance of AlgR: regulation of the *algD* promoter

As mentioned above, the release of AlgU to the cytoplasm increases its availability for activating the expression of the alginate biosynthetic enzymes encoded on the PA3540–3551 operon (Martin et al., 1993a; Devries and Ohman, 1994; Schurr et al., 1994). The alginate biosynthetic pathway was first discovered in the nitrogen-fixing diazotroph, *Azotobacter vinelandii* (Pindar and Bucke, 1975) and subsequently characterized in *P. aeruginosa*. In *P. aeruginosa*, the *algD* gene (PA3540) is the first ORF in an operon (PA3540–3551) comprised of 12 genes (*algD, alg8, alg44, algK, algE, algG, algX, algL, algI, algJ, algF*, and *algA*) encoding proteins involved in alginate biosynthesis, as well as its modification and export (Gacesa and Russell, 1990; Franklin et al., 2011; Okkotsu et al., 2013a). Transcription of the above genes is increased significantly in mucoid *P. aeruginosa*, leading to the over-production of alginate (Deretic et al., 1987a). Another gene, *algC* (PA5322), encoding a phosphomannomutase, is located distally to the *algD* locus, yet is still part of the alginate pathway (Zielinski et al., 1991).

The regulation of the *algD* promoter/alginate biosynthetic genes (PA3540–3551) is rather complex (Figure 2B). In addition to AlgU, the following proteins bind and regulate *algD* transcription: (a) AlgR (see below); (b) KinB/AlgB, a two component system where the AlgB (NtrC-family) response regulator positively regulates expression in a RpoN (σ^54^) dependent manner (Goldberg and Ohman, 1987; Wozniak and Ohman, 1991; Goldberg and Dahnke, 1992; Damron et al., 2012); (c) AmrZ, a positive regulator of the ribbon-helix-ribbon family of proteins (Baynham and Wozniak, 1996; Baynham et al., 1999, 2006; Pryor et al., 2012); (d) Integration Host Factor (IHF), a histone-like protein that binds to several regions on the promoter to induce DNA bending and activate transcription (Mohr et al., 1992; Toussaint et al., 1993); (e) AlgP (AlgR3), with a C-terminus histone-like element which also bends DNA and activates transcription (Deretic and Konyecsni, 1990; Deretic et al., 1992a); (f) the activator CysB, a LysR-like transcriptional regulator that is also a central regulator of cysteine metabolism (Delic-Attree et al., 1997); and (g) CRP (Vfr), a c-AMP dependent transcriptional regulator involved with the expression of several virulence genes (Devault et al., 1991). The reader is referred to extensive reviews for further details (Franklin et al., 2011; Okkotsu et al., 2013a).

AlgR was the first regulator discovered to be required for alginate over-production (Darzins and Chakrabarty, 1984). A *P. aeruginosa* chromosomal cosmid library was placed in chemically mutagenized non-mucoid strains, to screen for mucoid-rescue phenotypes (Darzins and Chakrabarty, 1984). A cosmid that restored alginate production in one particular mutant (strain Pa 8873) contained an ORF distal from the alginate biosynthetic genes (PA3540–3551). This 27.6 kDa protein was named AlgR (PA5261) for its ability to regulate alginate. AlgR contains an N-terminus with homology to CheY-like signaling transcriptional regulators (such as OmpR, NtrC) (Deretic et al., 1989), and a C-terminus with homology to LytR/YehT/AgrA family of DNA-binding transcriptional regulators (Galperin et al., 2001; Nikolskaya and Galperin, 2002; Sidote et al., 2008).

**Figure 3:**
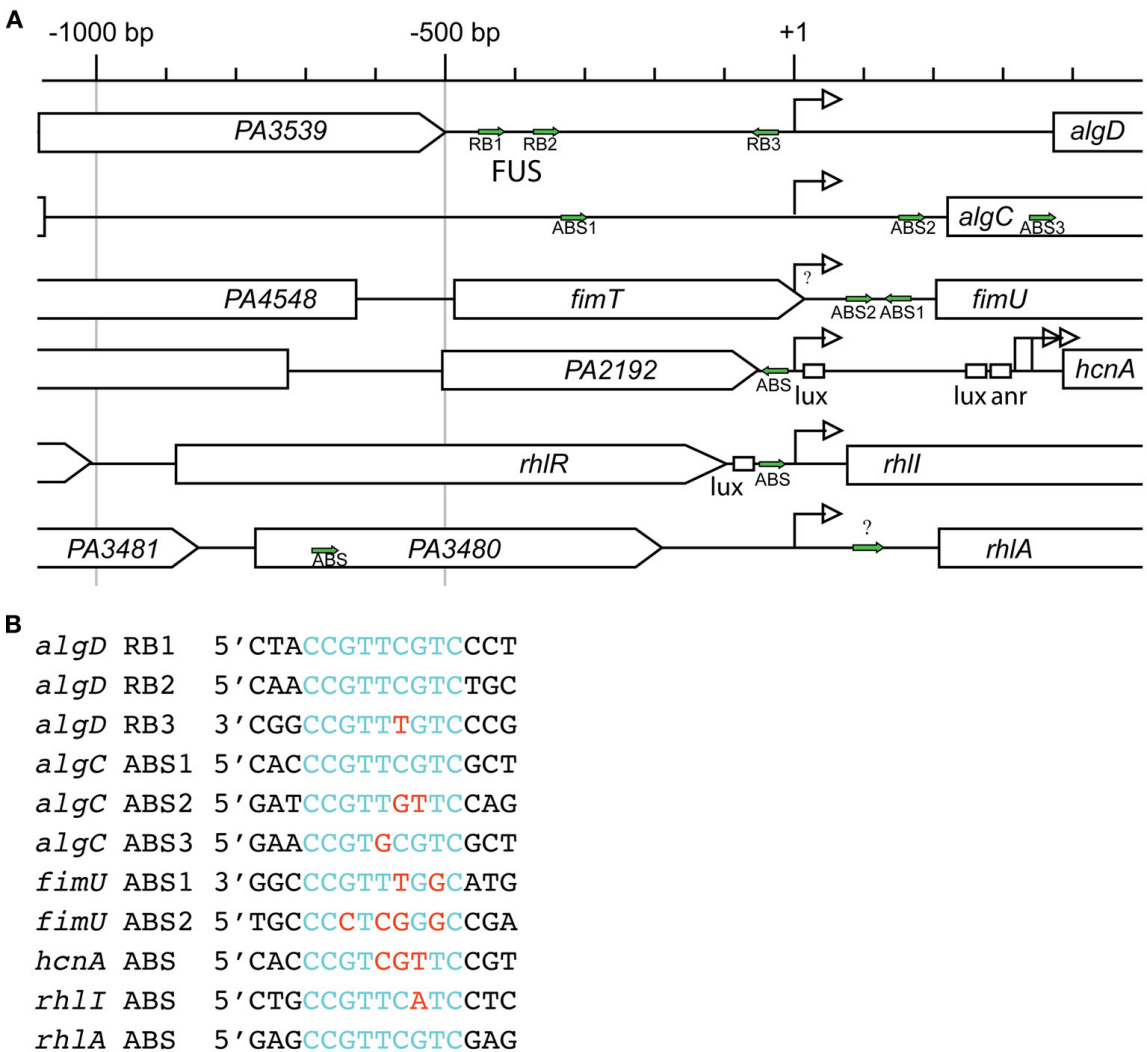
**AlgR regulated promoter complexity. (A)** Comparison of AlgR binding site locations within its regulated gene promoters. Gene names are labeled within arrowed boxes, and pointed end of boxes indicate open coding direction. Promoters are aligned to the gene's transcriptional start sites (+1). Identified AlgR-binding sites (ABS) are indicated by green arrows. Lux, LasR or RhlR binding box; anr, Anr binding box; bp, base pairs. **(B)** Comparison of AlgR binding sites consensus sequences. Red letters, different nucleotides compared to consensus sequence 5′-CCGTTCGTC-3′ found on *algD*-RB1 and *algD*-RB2; Blue letters, nucleotides identical to consensus sequence 5′-CCGTTCGTC-3′ found on *algD*-RB1 and *algD*-RB2.

Transcription of *algD* is dependent on AlgR, as *algR* deletion in phenotypically mucoid backgrounds abrogated *algD* promoter activity as well as, alginate production (Deretic et al., 1987a, 1989; Mohr and Deretic, 1990). AlgR regulates the *algD* promoter through three distinct binding sites, termed RB1, RB2, and RB3 (Figures 3A,B). Electrophoretic mobility shift assays (EMSA) and DNase I footprinting have demonstrated two AlgR binding sequences in a region −332 bp upstream of the transcriptional start site, named the far-upstream site (FUS). The binding sites, named *algD*-RB1 located at −470 bp, and *algD*-RB2 located at −394 bp upstream of the transcriptional start site, share a core 9 bp consensus sequence (5′-CCGTTCGTC-3′) (Kato and Chakrabarty, 1991; Mohr et al., 1991). Additionally, a third AlgR binding sequence, *algD*-RB3 (5′-CCGTTTGTC-3′) is located −45 bp upstream of the transcriptional start site in the opposite orientation (Kato and Chakrabarty, 1991; Mohr et al., 1991, 1992). The occupation of all three AlgR binding sites is required for maximal *algD* transcription.

Promoter analyses show that the FUS site is required for *algD* expression, as deletion of the FUS results in a 10-fold reduction in promoter activity (Deretic et al., 1987b; Mohr et al., 1990a). A study using a *algD* transcriptional reporter showed that deletion of *algD*-RB1 resulted in a 70% reduction, and deletion of both *algD*-RB1/*algD*- RB2 resulted in a 99% reduction in *algD* promoter activity (Mohr et al., 1990a). A mutation in *algD*-RB3 also reduced *algD* transcription by approximately 30% of the wild type promoter (Mohr et al., 1992). As these binding sites are located at relatively far distances, DNA looping is a proposed mechanism by which AlgR and the other transcriptional regulators affect the activity of the *algD* promoter in the mucoid background (May et al., 1991; Schurr et al., 1993) (Figure 2B).

Additionally, binding affinity of AlgR to these AlgR binding sequences is also a contributing factor to *algD* regulation. Affinity of AlgR toward the two AlgR binding sequences in the FUS is higher (*algD*-RB1, *K_d_* = 6.0 × 10^−8^; *algD*-RB2, *K_d_* = 7.2 × 10^−8^) than toward the *algD*-RB3 (*K_d_* = 3.7 × 10^−7^) (Mohr et al., 1992). The lower affinity is attributed to the single base difference within *algD*-RB3 (5′-CCGTTTGTC-3′, different base is underlined) compared to its consensus sequence (Mohr et al., 1992). Changing the sequence of *algD*-RB3 to a higher affinity sequence partially restored *algD* transcription, indicating that this third binding site is critical for modulating transcription (Mohr et al., 1992). Furthermore, overexpressing AlgR repressed *algD* transcription and alginate production, indicating that tight regulation of intracellular AlgR amounts is required for maximal *algD* promoter activity (Deretic and Konyecsni, 1989).

## AlgR regulation of the *algC* promoter

AlgR regulates the expression of another enzyme in the alginate biosynthetic pathway, AlgC, encoded by the *algC* gene (PA5322). AlgC is a phosphohexomutase enzyme that has dual phosphomannomutase/phosphoglucomutase activity (Regni et al., 2002). AlgC is a multifunctional enzyme, responsible for converting mannose 6-phosphate to mannose 1-phosphate (a key step in both alginate and Psl polysaccharide biosynthetic pathways), and converting glucose 6-phosphate to glucose 1-phosphate, to provide a precursor for the core region of lipopolysaccharide (LPS) as well as the sugar moiety for rhamnolipids (Goldberg et al., 1993; Coyne et al., 1994; Olvera et al., 1999).

The utilization of a cosmid library in conjunction with a chemically mutagenized strain library led to the discovery of *algC* (Darzins and Chakrabarty, 1984; Zielinski et al., 1991). The *algC* gene is distal to the *algD* operon. Nevertheless, AlgR still acts as an activator of *algC* transcription, as the activity of a *algC::lacZ* transcriptional reporter decreased four-fold in an *algR* deletion background (Zielinski et al., 1991). Like the *algD* promoter, the *algC* promoter contains three AlgR binding sequences, termed ABS1-3, (Zielinski et al., 1991; Fujiwara et al., 1993) albeit their positions relative to the transcriptional start sites are different from those found in *algD* (Figure 3A). The *algC*-ABS1 is located at −94 bp upstream, and *algC*-ABS2 is located at +163 bp downstream of the *algC* transcriptional start site. The *algC*-ABS3 is located +391 bp of the transcriptional start site, within the *algC* ORF. While *algC*-ABS1 (5′-CCGTTCGTC-3′) and *algC*-ABS3 (5′-CCGTGCGTC-3′) are higher affinity ABSs, *algC*-ABS2 (5 ′-CCGTTGTTC-3′) has two base pair changes as compared to the consensus sequence, and *algC*-ABS2 displays lower affinity to AlgR.

There is evidence that *algC*-ABS1 acts like a eukaryotic enhancer element (Fujiwara et al., 1993). Placing the *algC*-ABS1 to either +532 bp downstream or −432 bp upstream of the transcriptional start site did not generally affect *algC* transcriptional reporter activity, as both conditions resulted in transcriptional reporter activity that was 126 or 87% activity of wild type, respectively. Replacing *algC*-ABS1 with its reverse-complement also retained reporter activity (98% activity). Yet, there was a reduction to 0.5% activity when *algC*-ABS1 was deleted. Additionally, all three binding sites were required for maximal *algC::lacZ* expression (Fujiwara et al., 1993). In summary, these data support the idea that the occupation of all three AlgR binding sequences is required for optimal expression from the *algD* and *algC* promoters.

## Transcriptional regulation of *AlgR*

Concomitant with the activation of the *algD* promoter in mucoid *P. aeruginosa, algR* gene expression is also significantly increased (up to 50-fold) as compared to non-mucoid backgrounds (Deretic and Konyecsni, 1989; Kimbara and Chakrabarty, 1989; Deretic et al., 1990). In mucoid *P. aeruginosa, algR* expression is maximal at early stationary phase, coinciding with expression of several alginate biosynthesis genes including *algA, algC*, and *algD* (Leitao and Sa-Correia, 1995).

The region upstream of *algR* contains at least two transcriptional start sites (Martin et al., 1994; Wozniak and Ohman, 1994). AlgU acts on one of the promoters proximal (−73 bp) to the *algR* ATG. This proximal promoter contains an AlgU binding sequence (5′-GCACTT-N_17_-TCTGA) (Wozniak and Ohman, 1994; Firoved and Deretic, 2003). Therefore, as alluded to earlier, AlgR is part of the AlgU regulon and AlgR expression is responsive to membrane perturbations that result in the degradation of MucA, or through mutations in the *mucA, mucB*, or *mucD* genes (Figure 2A).

Another transcriptional start site, located at least 160 bp upstream of the *algR* ORF appears to be expressed constitutively (Mohr and Deretic, 1990; Mohr et al., 1990a); the expression from this *algR* promoter appears to not be affected by the mucoid status of the bacteria (Mohr et al., 1990a). This *algR* promoter is also independent of the nitrogen-responsive sigma factor RpoN (sigma-54) (Deretic et al., 1990; Mohr et al., 1990a), though, in one study, RpoN dependence was demonstrated in a *rpoN* gene deletion in a particular strain (Pa PAK-SN) (Kimbara and Chakrabarty, 1989). This *algR* promoter is responsive to increased osmolarity (Kimbara and Chakrabarty, 1989; Deretic et al., 1990; Mohr et al., 1990a) and nitrate (Deretic et al., 1990) in the mucoid background. However, the responsiveness is contingent on different *muc* alleles that result in the mucoid phenotype (Deretic et al., 1990). Incidentally, *algR* transcription is not responsive to changes in osmolarity or nitrate levels in the strain containing the *mucA22* allele (which is most commonly isolated from CF patients) (Deretic et al., 1990). The reason for these different responses is unclear at this time.

## AlgR orthologs in other bacteria

Inactivation of *algR* in mucoid *P. aeruginosa* isolates results in a loss of mucoidy (Deretic et al., 1990; Wozniak and Ohman, 1994). The *algR* gene is also required for alginate production in other *Pseudomonas* species. *P. syringae* is a plant pathogen responsible for dieback and canker disease in ornamental pears. Similar to *P. aeruginosa*, alginate production in *P. syringae* requires *algR* and the expression of the *algD* operon and *algC* gene. Alginate provides a medium for nutrient accumulation, water absorption and water retention, thus preventing desiccation of the organism (Denny, 1995). The *algR* gene is a virulence determinant for *P. syringae* pv. syringae FF5, as deletion of *algR* results in decreased necrosis in a tobacco leaf model of infection (Penaloza-Vazquez et al., 2004). However, consensus *algR* binding sites are absent on the *algD* promoter, and AlgR is not involved with the transcription of the *algD* gene (Fakhr et al., 1999). Instead, AlgR and the sigma factor RpoN, regulate *algC* transcription, as deletion of *algR* or *rpoN* results in decreased *algC* expression (Penaloza-Vazquez et al., 2004). Similarly, in *P. putida* WCS358, its AlgR homolog called PprA, activates *algC* but not *algD* transcription. Additionally, complementation with the *pprA* gene in a non-mucoid *P. aeruginosa* containing a *algR* deletion allele (Pa 8852) restores mucoidy (Venturi et al., 1995), supporting the idea that PprA is a functional ortholog of AlgR.

Through Southern hybridization, *algR* has been detected in *A. vinelandii, Azomonas macrocytogenes, Xanthomonas campestris*, and *Serpens flexibilis* (Fialho et al., 1990). However, *algR* has only been examined in *A. vinelandii* thus far. *A. vinelandii* is a free-living (unassociated with rhizobium), nitrogen-fixing, soil-borne organism, which has an alginate-encapsulated cyst as part of its lifecycle. Alginate serves to protect the cyst from desiccation, as well as to protect its nitrogenases from excess exogenous oxygen (Clementi, 1997). The *A.v.* AlgR ortholog, which is 79% identical to *P.*a. AlgR is required for encystment, as a *algR* mutant of *A. vinelandii* displays a 1000-fold reduction in its resistance against desiccation (Nunez et al., 1999)*. A.v.* AlgR does not directly regulate the transcription of *A.v.-algD*, rather, alginate production is controlled through *A.v.*-AlgR activation of *A.v.-algC* (Nunez et al., 1999). These data suggest that the role of AlgR on exopolysaccharide production is conserved across genera, but the exact genes that it regulates are species-specific.

## AlgR protein

**Figure 4:**
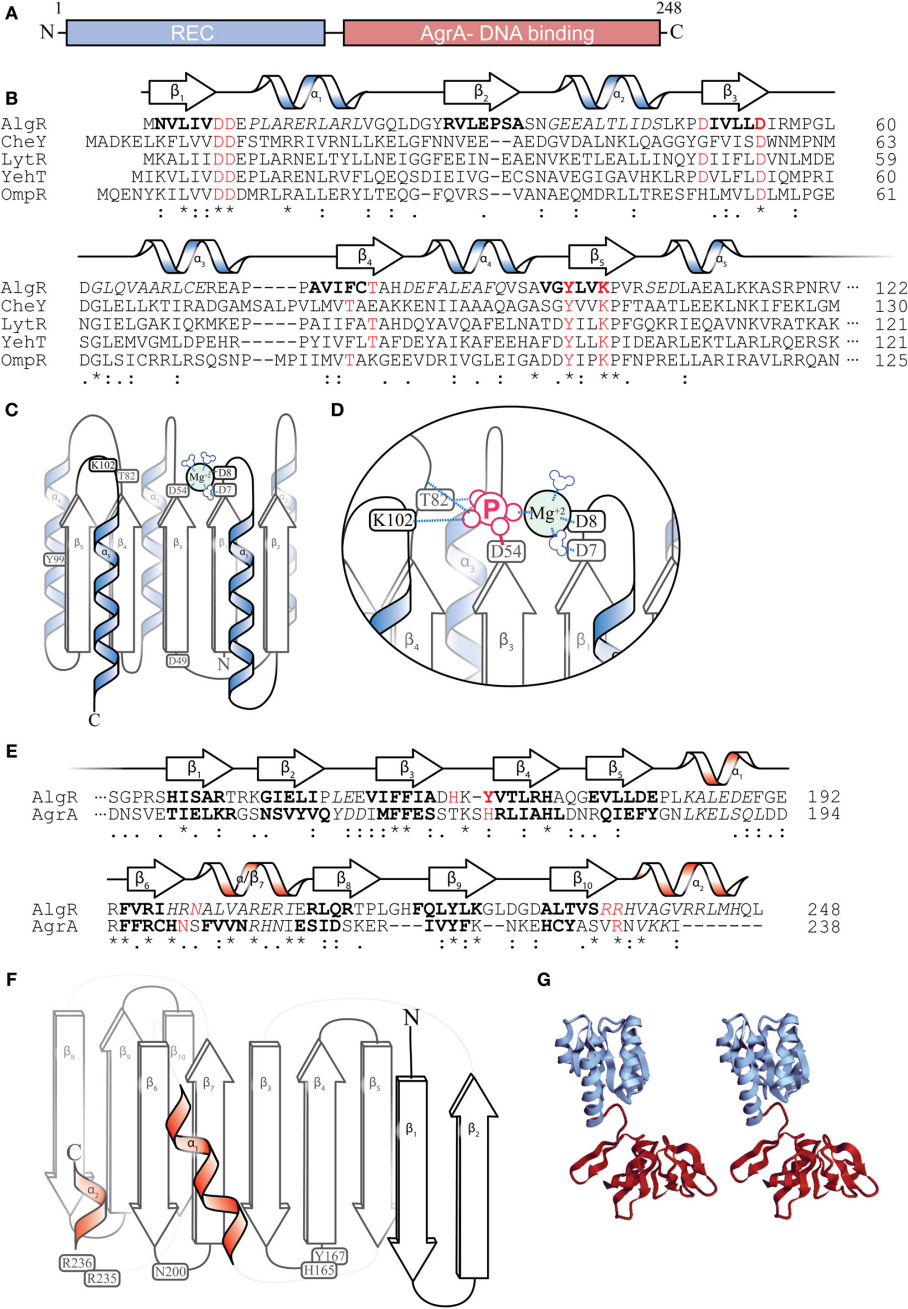
**AlgR Structural predictions. (A)** Diagram depicting two major domains of the 249 amino acid AlgR polypeptide. **(B)** A ClustalW sequence alignment of the amino acid sequence of the N-terminus CheY-like receiver (REC) domain of AlgR and its orthologs. “^*^” = identical amino acids, “:” = conserved substitutions, “.” = semi-conserved substitutions. Red color denotes select highly conserved amino acids, most with functional importance for phosphorylation, italics indicate alpha helices, bold indicates beta sheets. The predicted secondary structure of AlgR according to Phyre2 is located above the sequence alignment. α = alpha helix, β = beta sheet. **(C)** An illustrated model depicting the location of conserved amino acids on the secondary structures of the REC domain of AlgR. Aspartate 7, 8, and 54 of CheY-like domains coordinate a magnesium ion, and are required for aspartyl-phosphate transfer from histidine kinase. **(D)** Once phosphorylated, residues threonine 82 and lysine 102 interact with phosphate ion, changing the conformation of the REC domain. **(E)** A ClustalW sequence alignment of AlgR and AgrA, with same annotation as **(B)**. Red color denotes select conserved amino acids that are required for DNA interaction in AgrA, and may be required for AlgR interaction with DNA. **(F)** An illustrated model depicting the location of conserved amino acids on the secondary structures of the LytR/AgrA DNA-binding domain of AlgR. **(G)** a stereoscopic view of the full AlgR protein modeled, using CheY structure and *S. aureus* AgrA DNA binding domain.

According to protein structure predictions, AlgR has two major structural domains: (i) an N-terminal CheY-like receiver (REC) domain (Deretic et al., 1989; Whitchurch et al., 2002; Kelley and Sternberg, 2009); and (ii) a carboxyl DNA-binding domain of the LytR/YehT/AgrA family of transcriptional regulators (Galperin et al., 2001; Nikolskaya and Galperin, 2002; Sidote et al., 2008) (Figures 4A,G). CheY is a well-studied signaling protein involved with the flagellar motor switch complex (Bren and Eisenbach, 2000). Phosphorylation of a conserved aspartate (Asp57) residue in CheY by its histidine kinase, CheA, leads to a clockwise rotation of the flagella. CheY and CheY-like domains contain a set of 5 alternating β-pleated sheets and α-helices (Figure 4B) (Bourret, 2010). The AlgR-REC domain contains the residues required for aspartyl- phosphorylation. While a crystal structure for AlgR has not been determined, various inferences could be made from its conserved residues. For instance, Asp7, Asp8, and Asp54 potentially form an acidic pocket that coordinates a divalent cation (Mg^+2^), which is required for catalyzing the phosphorylation of Asp54 (Figures 4C,D) (Whitchurch et al., 2002). Like many CheY-like domain containing response regulators (RR) (Lukat et al., 1992), AlgR is able to catalyze its own auto-phosphorylation in the presence of high-energy phospho-donors such as carbamyl phosphate and acetyl phosphate (Deretic et al., 1992b; Okkotsu et al., 2013b). A conservative substitution of the Asp54 residue of AlgR to an asparagine (D54N) eliminates its *in vitro* phosphorylation by an *E. coli* derived CheA HK, indicating that, like other RRs, this Asp54 residue is required for phospho-transfer (Whitchurch et al., 2002). Upon interaction with and phosphorylation by its cognate histidine kinase, the Asp54 could then covalently bind to phosphate and introduce a negative charge within the acid pocket of AlgR, displacing the Mg^+2^ ion (Figure 4D). Oxygen groups on the negative charged phosphate are proposed to interact with Thr82 (hydrogen bonding) and Lys102 (salt bridge) (Figure 4D), which would alter the conformation and stabilize the AlgR REC domain.

The C-terminus of AlgR contains a DNA-binding domain in the LytR/YehT/AgrA family of transcriptional regulators (Galperin et al., 2001; Nikolskaya and Galperin, 2002; Sidote et al., 2008). This family is very uncommon, as it represents only 3% of all sequenced response regulators in bacteria. In contrast, winged-helix and helix-turn-helix domains are present in 30.1 and 16.9% of response regulators, respectively (Galperin, 2010). Helix-turn-helix (HTH) (e.g., NtrC) (Pelton et al., 1999) or winged-helix (e.g., OmpR) (Martinez-Hackert and Stock, 1997) motifs (Galperin, 2010) contain alpha-helices that embed in the major grooves of the DNA to make specific hydrogen bond contacts with the recognition sequence. On the other hand, the LytR/YehT/AgrA/AlgR family of transcriptional regulators utilizes unstructured loop regions to recognize DNA sequences (Sidote et al., 2008).

Though the crystal structure of AlgR has not been solved, secondary structure prediction based on the Phyre2 protein fold recognition server (http://www.sbg.bio.ic.ac.uk/phyre2) using the last 139 a.a. of AlgR (DNA-binding region) predicts that AlgR carboxyl terminus has at least 9 beta-pleated sheets, and demonstrates a 100% precision (*E*-value 2.4e–11) with the AgrA C-terminus domain (Figures 4E,F) (Sidote et al., 2008). AgrA is a *Staphylococcus aureus* RR that (together with AgrC HK) regulates the *agr* (*accessory gene regulator*) locus in *S. aureus*. The *agr* locus encodes a set of regulatory RNAs (RNAIII) that control *S. aureus* virulence factors in a temporal, cell-density (quorum) dependent manner (Novick, 2003). In AgrA, flexible loops (unstructured regions) between pairs of beta-pleated sheets make 2 or 3 specific contacts in successive major grooves (Sidote et al., 2008). The residues that make these interactions in AgrA are His169, Asn201, and Arg233. AlgR also has a conserved Asn200 and a pair of arginine residues (Arg235 and Arg236) that may correspond to those found in AgrA (Figure 4E). In addition to AgrA, another LytR-type response regulator from *Bacillus cereus* has been submitted to the RCSB Protein Data Bank (Osipiuk et al., to be published, PBD id: 3d6w) and will give additional insight into the structure of this family of regulators.

The LytR/YehT/AgrA/AlgR transcriptional regulators commonly affect the expression of virulence factors (Nikolskaya and Galperin, 2002). Some of the more well-studied regulators in this family include YehT of *E. coli* (Kraxenberger et al., 2012), AgrA of *S. aureus* (Koenig et al., 2004; Sidote et al., 2008), FsrA of *Enterococcus faecalis* (Del Papa and Perego, 2011), VirR of *Clostridium perfringens* (Cheung and Rood, 2000; Cheung et al., 2004), and PlnC and PlnD of *Lactobacilus plantarum* (Risoen et al., 1998, 2001; Straume et al., 2009). A common characteristic of these regulators is their mode of binding to DNA (Sidote et al., 2008). That is, they bind sequences that are between 8 and 13 bp long, arranged as two imperfect direct repeats approximately 12 bp apart (Risoen et al., 2001; Cheung et al., 2004; Koenig et al., 2004; Belete et al., 2008; Del Papa and Perego, 2011). The exact spacing between the direct repeats is crucial for gene transcription, though the molecular basis for this exact spacing is unknown (Sidote et al., 2008). According to the crystal structure of AgrA, large bends in the DNA are created to accommodate protein binding as direct repeats (Sidote et al., 2008). Interestingly, AlgR binding sites are not necessarily arranged as direct repeats separated by 12 bp, as seen in the *algD, algC*, and other AlgR-regulated promoters (Figures 3A,B). The reason for this is unclear at the moment, as further studies on AlgR are required.

## The putative histidine kinase for AlgR is encoded by *AlgZ/FimS*

AlgR belongs to the AlgZ/FimS-AlgR two-component regulatory system (TCS) (Deretic et al., 1989; Galperin et al., 2001). TCSs are bacterial sensing systems that couple environmental stimuli to adaptive responses (Stock et al., 2000; Gao and Stock, 2009). TCSs typically consist of a homodimeric histidine kinase (HK, sensor component) and a response regulator (RR, response component). AlgR's putative cognate histidine kinase is located upstream of the *algR* gene (Figure 2A), and is called *algZ/fimS* (PA5262) (Whitchurch et al., 1996; Yu et al., 1997). Another protein named AlgZ also exists in the literature for an AlgU/T-dependent DNA binding protein whose name has been changed to AmrZ to avoid future confusion (Baynham and Wozniak, 1996; Baynham et al., 1999, 2006). The ORF of *algZ/fimS* is 1077 nucleotides, encoding a 40.1 kDa polypeptide. However, the amino acid sequence of AlgZ does not show similarity to most histidine kinases. Accordingly, it belongs to a subset of non-canonical histidine kinases called the “unorthodox family of histidine kinases” (Kim and Forst, 2001). Other members of this atypical family, including LytS (Brunskill and Bayles, 1996), YehU (Kraxenberger et al., 2012), and the proposed AlgZ in the AlgZR system in *Acinetobacter baumanii* (*ABAYE 3510*) (Adams et al., 2008), are missing motifs normally required for ATP-mediated autophosphorylation.

A prototypical HK contains a membrane-bound sensor domain unique to the signals being perceived. Using secondary structure prediction analyses (Kyte and Doolittle, 1982; Engelman et al., 1986) and software (SOSUI, TMHMM 2.0) (Hirokawa et al., 1998; Sonnhammer et al., 1998; Krogh et al., 2001), the first 150 amino-terminal amino acids of AlgZ are predicted to fold as four transmembrane helices. This arrangement is similar to many membrane anchored histidine kinases, and strongly suggests AlgZ is localized to the membrane. However, the functional role of AlgZ's sensor domain is currently unknown, and is an area of active study by our lab.

Histidine kinases typically consist of a DHp domain (Dimerization and Histidine phosphotransfer) and a CA domain (Catalytic and ATP binding) (Kelley and Sternberg, 2009; Kallberg et al., 2012). In general, the DHp and CA domains house conserved motifs, termed “boxes” that are required for proper kinase function, known as the H, N, G1, F, and G2 boxes. The DHp domain accommodates the H box, a 9 amino acid sequence containing the phosphorylatable histidine central to phosphotransfer. The CA domain coordinates an ATP molecule through the N, G1, F, and G2 boxes to mediate the phosphotransfer reaction. The N box forms part of the ATP binding pocket, and the G1, F, and G2 boxes are found in a flexible lid-like structure which coordinates the ATP molecule within the pocket (Stock et al., 1989; Parkinson and Kofoid, 1992).

Secondary structure prediction software (Raptor X and Phyre2) now suggest that the cytoplasmic domain of AlgZ (amino acids 151–358) contains the kinase, but it was only after the characterization of its orthologs (e.g., *S. aureus* LytS and *E. coli* YehU) that AlgZ was considered to be part of this class of HKs. Part of the difficulty in categorizing AlgZ was due to the lack of sequence identity within the carboxyl-terminus. While the H box, having the sequence RPHFLFNSL (blue indicates conserved histidine residue, underline indicates canonical homology), shows reasonable identity among this group of proteins, the N box, LQPLLENALIYG (blue indicates conserved asparagine residue, underline indicates canonical homology) only retains 5 of the 12 amino acids found in the canonical sequence. The obvious similarities between AlgZ and canonical histidine kinases are limited to these two boxes, as AlgZ lacks identifiable G1, F, and G2 boxes that make up the ATP-coordinating flexible lid. Even among its most similar orthologs (e.g., LytS or YehU), AlgZ is unique as LytS contains both G boxes, and YehU contains one of the two. It is possible that AlgZ may bind and coordinate ATP in an atypical manner, or bind a related molecule like GTP, as recently identified in the *Bacillus anthracis* histidine kinase (BA2291) (Scaramozzino et al., 2009).

Because AlgR was first identified as a two-component regulator of alginate production, it was expected that its putative histidine kinase would play a regulatory role in alginate production (Deretic et al., 1989). However, as it turns out, AlgR phosphorylation may not be required for alginate production. Expression of the *algR*D54N allele (expressing the phospho-defective AlgR protein) in the mucoid strain FRD1 does not affect alginate production (Ma et al., 1998). In fact, deletion of *algZ/fimS* in PAO1, or its mucoid derivative PAO568, resulted in a two-fold increase in alginate production (Yu et al., 1997), suggesting that AlgR phosphorylation may not effect, or even inhibit alginate production in the mucoid background. There is currently limited characterization of AlgZ; neither direct interaction of AlgZ and AlgR, nor phospho-transfer between AlgZ and AlgR have been demonstrated. Therefore, its proposed role as the cognate HK for AlgR has been inferred from genetic evidence on its regulation of the *fimU* and *hcnA* promoters (Belete et al., 2008; Cody et al., 2009). As described below, twitching motility, requires AlgR and its HK, AlgZ/FimS (Whitchurch et al., 1996).

## AlgZ/R controls type IVa pili at the *fimUpilVWXY1E* promoter

The discovery that AlgZ/FimS and AlgR are required for twitching motility (Whitchurch et al., 1996) prompted a search for AlgR regulated genes that control twitching motility (Lizewski et al., 2004). Twitching motility is mediated by type IVa pili. AlgR regulates type IVa pili function and twitching motility through transcriptional activation of the *fimUpilVWXY1Y2E* operon, as an *algR* deletion abrogated expression from this operon (Lizewski et al., 2004; Belete et al., 2008). Additionally, trans-complementation of the *fimU* operon into a PAO1 Δ*algR* strain restored twitching motility, indicating AlgR controls twitching motility by regulating the expression of the prepillin genes (Lizewski et al., 2004). The *fimUpilVWXY1Y2E* genes encode prepillins (FimU, PilV, PilW, PilX, PilE) that incorporate into the type IVa pili, and a calcium-dependent retraction protein (PilY1). The *pilY2* ORF is located in this operon but is proposed to be a pseudogene (Giltner et al., 2010).

All of the genes encoded in the operon *fimUpilVWXY1Y2E* contain a hydrophobic N-terminus leader sequence required for translocation to the periplasm for type IVa pili assembly (Russell and Darzins, 1994; Alm and Mattick, 1995; Alm et al., 1996; Alm and Mattick, 1996) FimU, PilV, PilW, PilX, and PilE incorporate and create a complex with the pilus fiber (Giltner et al., 2010). The PilY1 protein, encoded by *pilY1* gene, is an ortholog of the *Neisseria gonorrhoeae* PilC adhesin protein (Rudel et al., 1995). PilY1 appears to be a multi- functional protein: *pilY1* encodes a surface and outer-membrane associated integrin-binding protein (Heiniger et al., 2010; Johnson et al., 2012) that can inhibit PilT-mediated pilus retraction (Orans et al., 2010). Surface expression of type IVa pili requires a proper stoichiometric expression of the prepillins; the interaction/compatibility of the FimU, PilV, PilW, PilX, PilY1, and PilE with other pilus assembly components dictates the efficiency of pilus assembly and disassembly (Giltner et al., 2010, 2011). Therefore, the negative twitching motility phenotype observed by a strain containing a Δ*algR* allele is proposed to be due to inefficient pilus assembly and extrusion.

AlgR phosphorylation is also required for *fimUpilVWXY1Y2E* operon activation. A strain lacking *algZ/fimS* (Whitchurch et al., 1996) or expressing a phospho-defective AlgR D54N loses twitching motility (Whitchurch et al., 2002) and expresses these prepilin genes at a lower level (Belete et al., 2008). More recently, a strain that expressed a gene encoding a phospho-mimetic isoform of AlgR (*algR*D54E) activated *fimU* transcription, while *algZ* deletion abrogated *fimU* transcription (Okkotsu et al., 2013b). As demonstrated by DNaseI Footprinting and EMSA, AlgR binds to an intergenic region between *fimU* and the upstream gene *fimT* at *fimU*-ABS1 (5′-CCGTTTGGC-3′), as well as *fimU*-ABS2 (5′-CCCTCGGGC-3′) (Figures 3A,B) (Belete et al., 2008). *In vitro*, phosphorylated AlgR displayed increased affinity for the *fimU* promoter region as compared to un-phosphorylated AlgR (Okkotsu et al., 2013b). Phosphorylation of the REC domain from other LytTR response regulators such as AgrA (Koenig et al., 2004) and both PlnC and PlnD (Risoen et al., 2001) also increased affinity for DNA. Together, these data indicate that AlgR phosphorylation by AlgZ increased AlgR affinity to the *fimU* promoter and activated its transcription. Though the signal for the AlgZ sensor kinase has not been determined, the signal, via AlgR phosphorylation, activates twitching motility by increasing the expression of these prepilins.

## AlgR and the Rhl quorum sensing system

*P. aeruginosa* harboring the phosphorylation defective *algR*D54N allele was observed to have a defect in biofilm formation, as well as twitching motility (Whitchurch et al., 2002). Microbial biofilms are highly complex, multicellular communities. Organisms are embedded in a matrix that consists of proteins, polysaccharides, and extracellular DNA (eDNA). There are multiple steps in biofilm maturation: (1) initial attachment, (2) microcolony formation, (3) maturation, and (4) dispersion (Sauer et al., 2002; Stoodley et al., 2002). There is substantial evidence that quorum sensing is required for biofilm formation and virulence, both *in vitro* and *in vivo* (Davies et al., 1998; Rumbaugh et al., 2000; Bjarnsholt and Givskov, 2007; Murray et al., 2007). Quorum sensing is an auto-induction system that monitors cell density through the export of small molecules (Fuqua et al., 1994; Seed et al., 1995). *P. aeruginosa* has three main quorum sensing systems- the Las, Rhl, and PQS systems. The three systems, and the virulence factors they regulate are required for biofilm formation.

The LasI-LasR (Fuqua et al., 1994; Seed et al., 1995), and RhlI-RhlR (Brint and Ohman, 1995; Ochsner et al., 1995) quorum sensing pairs are LuxI-LuxR type regulators, where the LuxI component (LasI or RhlI) synthesize their respective N-acyl homoserine lactone (AHL) molecules (Passador et al., 1993; Winson et al., 1995), and the LuxR-type HTH-containing DNA-binding regulators (LasR and RhlR) sense the signals. LasI synthesizes N-(3-oxo-dodecanoyl)-L-homoserine lactone (3-oxo-C12-HSL), and RhlI synthesizes N-butanoyl-L-homoserine lactone (C4-HSL) from S-adenosylmethionine (SAM) and acyl-acyl carrier protein (ACP) from fatty acid biosynthesis (Parsek et al., 1999; Hoang et al., 2002). The LasR or RhlR regulators bind their respective signaling molecules, dimerize, and bind to lux-box sequences of their promoters to activate DNA transcription.

The PQS system produces 2-heptyl-3-hydroxy-4-quinolone (PQS) (Pesci et al., 1999) through a biosynthetic pathway encoded by the genes *pqsABCDE* and *pqsH* (D'Argenio et al., 2002; Gallagher et al., 2002; Deziel et al., 2004). These genes are also responsible for the production of up to 55 types of 4-hydroxyl-2-alkylquinolines (HAQs) (Lepine et al., 2004) that are similar to PQS and have antimicrobial properties (Lightbown and Jackson, 1956; Machan et al., 1992; Mahajan-Miklos et al., 1999). PQS induces the DNA-binding capacity of the LysR-type transcriptional regulator, PqsR/MvfR, to activate both the expression of the *pqsABCDE* (Wade et al., 2005) genes for auto-regulation, as well as genes responsible for the production of other HAQs (*phnAB*)(Deziel et al., 2004).

The interaction among the three QS circuits is complex. Studies have revealed a large amount of cross-talk among the three circuits (Dekimpe and Deziel, 2009) where LasR activates the *pqsR/mvfR* gene (Xiao et al., 2006), while RhlR represses *pqsABCDE* transcription (McGrath et al., 2004; Xiao et al., 2006). PQS and PqsR/MvfR can activate the Rhl quorum sensing system independently of the Las system (McKnight et al., 2000; Diggle et al., 2003) as well as virulence factors under RhlI/RhlR control (Deziel et al., 2005; Xiao et al., 2006). Adding to the complexity is an orphan regulator, QscR, which responds to 3-oxo-C12 HSL and represses quorum-sensing regulated factors including phenazine and hydrogen cyanide production, *rhlI/rhlR* and *lasI* expression (Chugani et al., 2001; Ledgham et al., 2003; Fuqua, 2006).

Deleting *algR* caused a defect in a 6-day biofilm, that was restored by *algR* complementation on a plasmid (Morici et al., 2007). Additionally, 765 genes were differentially regulated at least two-fold in a 6-day biofilm when PAO1 was compared to its isogenic *algR* deletion strain by global transcriptome analysis (Morici et al., 2007). According to this comparison, the majority of genes that showed differential expression included those in the Rhl quorum sensing system (Morici et al., 2007). Microarray analysis showed that AlgR repressed *rhlABC, hcnAB, lecB*, and genes in the biosynthetic pathway for pyocyanin production (*phzC2-G2*). As these genes are under the control of the Rhl quorum-sensing system, it was surmized that this regulation was through the *rhlI* gene, encoding the C4-homoserine lactone (HSL) autoinducer synthase. AlgR repressed *rhlI* transcription and bound the *rhlI* promoter *in vitro* directly at the *rhlI*-ABS (5′-CCGTTCATC-3′), located −28 bp upstream of the transcriptional start site (Figures 3A,B). Deletion of the AlgR binding site in the *rhlI* promoter showed a similar biofilm growth defect as in the *algR* deletion strain, providing additional evidence that AlgR suppressed the quorum sensing system at *rhlI* (Morici et al., 2007).

Another set of genes under the exclusive control of the Rhl QS system and highly regulated by AlgR, are the *rhlAB* and *rhlC* genes (Ochsner and Reiser, 1995; Croda-Garcia et al., 2011). These genes, encode key enzymes that are important for the production of the surface-active amphipathic glycolipids called rhamnolipids. Rhamnolipids are important wetting agents for group-coordinated swarming motility (Deziel et al., 2003), normal biofilm formation (Davey et al., 2003; Espinosa-Urgel, 2003), and are a virulence factor (McClure and Schiller, 1996; Jensen et al., 2007). In a biofilm, rhamnolipids are required for microcolony formation (Pamp and Tolker-Nielsen, 2007), fluid channel formation (Davey et al., 2003), and bacterial dispersion from a mature biofillm (Boles et al., 2005). The Δ*algR* mutant was unable to form structured biofilms, and instead, developed into a flat mat of cells over the course of 6-days (Morici et al., 2007). Both *rhlA* promoter activity and rhamnolipid production increased in an *algR* deletion strain indicating that AlgR represses *rhlA* in biofilms. Additionally, AlgR bound to the *rhlA* promoter *in vitro*, at *rhlA*-ABS1 (5′-CCGTTCGTC-3), located −702 bp upstream of the *rhlA* transcriptional start site (Figures 3A,B). Therefore, the biofilm defect in the Δ*algR* strain was attributed to an increase in rhamnolipid production that subsequently disrupted the biofilm structure.

More recently, it was demonstrated that over the course of an 11 day biofilm in non-mucoid (PAO1) and mucoid (PA17) strains, *algR* expression is negatively correlated with *rhlA* and *rhlB* expression. Specifically, a decrease in *algR* transcription in PAO1 at later time points (day 7 and beyond) was correlated with increased *rhlA* and *rhlB* transcription, where biofilm dispersion would occur (Wang et al., 2013a). It has also been demonstrated that AlgR phosphorylation modulates rhamnolipid production, as a phospho-defective isoform (*algR*D54N) was unable to express rhamnolipids, while a phospho-mimetic isoform (*algR*D54E) was able to express rhamnolipids. In support of this, rhamnolipid-dependent swarming motility was affected by the *algR*D54N allele, and complemented with a strain expressing the *rhlAB* genes (Okkotsu et al., 2013b). These results indicate that AlgR phosphorylation adds yet another level of regulation to rhamnolipid production. As mentioned above, type IVa pili expression and function (through the *fimUpilVWXY1E* genes) are dependent on AlgR (Lizewski et al., 2004). Type IVa pili are required for biofilm formation, as it is involved with initial micro-colony formation (O'Toole and Kolter, 1998; Heydorn et al., 2002). Type IVa pili also play a role in stalk and cap formation during biofilm establishment (Klausen et al., 2003; Wang et al., 2013b). Together, these results support an idea the AlgR levels may act to coordinate rhamnolipid production and twitching motility, possibly to aid in either early colonization or later biofilm dispersion.

## AlgR represses the expression of genes involved with anaerobic metabolism

Defining the AlgR regulon is of great interest; in addition to regulating alginate production in mucoid *P. aeruginosa*, type IVa pili mediated twitching motility, and the Rhl quorum sensing and rhamnolipid production in non-mucoid cells, AlgR likely has an even larger role in the organism's physiology. By comparing an *algR* deletion strain to its parental wild type PAO1 strain, it was demonstrated that AlgR is involved with regulating hundreds of genes in logarithmic, stationary, and biofilm modes of growth (Lizewski et al., 2002; Morici et al., 2007), and regulating the expression of at least 47 proteins during logarithmic growth (Lizewski et al., 2002). In all, 95 genes during mid-log phase and 59 genes during stationary phase were differentially regulated two-fold or greater, and 885 genes were differentially expressed when *algR* was overexpressed (Lizewski et al., 2004). While the majority of genes regulated by AlgR in mid-log and stationary phase encode hypothetical proteins, many of the known genes indicate AlgR may be involved with repressing anaerobic metabolism genes.

The transcriptional regulator, Anr (anaerobic regulation of arginine catabolism and nitrate reduction), is a ortholog of *E. coli* FNR, and regulates the anaerobic denitrification system (Sawers, 1991; Ye et al., 1995; Winteler and Haas, 1996). Anr is a homodimeric, [4Fe-4S]^+2^ cluster-containing transcriptional regulator of the Fnr-Crp family. Anr is activated at low oxygen concentrations; increased oxygen can dissociate the homodimer and inactivate Anr (Yoon et al., 2007). During anaerobic growth, Anr, along with Dnr (a NO-sensing, heme-containing transcriptional regulator) and NarXL (NO^−^_3_ sensing TCS) regulate denitrification, and genes required for fermentation of arginine and pyruvate (Zimmermann et al., 1991; Castiglione et al., 2009).

According to global transcriptional analysis, there is an overlap between Anr-regulated genes and AlgR-repressed genes (Lizewski et al., 2004). Some of these include: (i) *arcA* (PA5171), part of the *arcDABC* operon encoding anaerobic arginine deiminase enzymes, which catabolizes arginine to generate ATP through substrate level phosphorylation under anaerobic conditions (Vander Wauven et al., 1984; Galimand et al., 1991); (ii) *hemN* (PA1546), which encodes an oxygen-independent copropophyrinogen III oxidase important for heme biosynthesis under anaerobiosis (Rompf et al., 1998) and essential for anaerobic growth (Filiatrault et al., 2006); (iii) *ccoP2* (PA1555), *ccoO2* (PA1556), and *ccoN2* (PA1557), ccb_3_-2 type cytochrome oxidase components expressed at low oxygen concentrations (Comolli and Donohue, 2004); (iv) *oprG* (PA4067), which is an outer-membrane diffusion-driven specific transporter of hydrophobic molecules whose expression increases under anaerobic conditions in the presence of iron (McPhee et al., 2009; Touw et al., 2010), and; (v) *hcnB* (PA2194), a gene encoding hydrogen cyanide synthase responsible for the production of HCN, and a possible mediator of anaerobic growth (Zimmermann et al., 1991; Gallagher and Manoil, 2001; Cody et al., 2009).

There is substantial evidence to suggest that *P. aeruginosa* prefers to respire under microaerobic conditions (Schreiber et al., 2007). The CF lung is a low oxygen or anaerobic environment (Su and Hassett, 2012), and macrocolonies of *P. aeruginosa* found in the intraluminal space contain very low oxygen (2.5 mmHg, compared with 180 mmHg in the bronchial lumen) (Worlitzsch et al., 2002). Additionally, according to a survey of *P. aeruginosa* isolates from CF patients over time, organisms adapted for anaerobic metabolism are selected for during chronic infection (Hoboth et al., 2009). When “hypermutator” (late-CF infection) isolates were compared with their isogenic “non-mutator” (early-CF infection) isolates from the same patients, gene expression and/or protein production of *anr, arcDABC, oprG*, and *hemN* were increased in the chronic isolates as compared to the early isolates (Hoboth et al., 2009). Based on these results and the microarray data, AlgR appears to repress genes that are favorable for anaerobic growth in PAO1 when the organism is growing aerobically in logarithmic phase.

## AlgR and hydrogen cyanide production

Among AlgR regulated genes within the Anr regulon, the *hcnABC* (PA2193–2195) genes are the best studied. Hydrogen cyanide (HCN) is a volatile, highly reactive molecule that inhibits respiratory cytochromes and metalloenzymes. HCN is produced primarily at microaerobic conditions at high-cell density (Castric, 1983). HCN production may serve to adapt *P. aeruginosa* to low oxygen environments by shutting down its own low-affinity cytochrome oxidases (Williams et al., 2007). *P. aeruginosa* resists self-poisoning by expressing cyanide-insensitive cytochromes (CIO) (Cunningham and Williams, 1995; Cunningham et al., 1997).

The *hcnABC* genes encode the synthase complex that creates HCN from glycine by oxidative decarboxylation (Castric, 1977; Laville et al., 1998). Expression of the *hcnC* gene contributes to killing in *Drosophila melanogaster* (Broderick et al., 2008) and *Caenorhabditis elegans* (Gallagher and Manoil, 2001) models of infection. HCN production may also be a clinically relevant virulence factor in CF patients. Cyanide in the CF sputum is associated with decreased pulmonary function as determined by forced expiratory volume (FEV) and forced vital capacity (FVC) (Ryall et al., 2008). Additionally, increased cyanide production was observed by 21 mucoid clinical CF isolates as compared to non-mucoid isolates from the same patient and sputum sample (Carterson et al., 2004). Detection of cyanide was proposed to be a potential *in vivo* biomarker for virulent, micro-aerobically growing *P. aeruginosa* (Sanderson et al., 2008). However, recent data suggest that HCN production may not be a biomarker for *P. aeruginosa* infection, as increased HCN levels in the bronchoalveolar lavage of CF patients was attributed to neutrophilic inflammation (Stutz et al., 2011), and HCN was also generated by salivary peroxidase in the oral cavity (Dummer et al., 2013).

Regulation of *hcnABC* expression is complex. The *hcnA* promoter is controlled by a number of factors, including Anr (Zimmermann et al., 1991), small RNA regulatory proteins GacA/RsmA (Pessi and Haas, 2001; Lapouge et al., 2007), quorum sensing regulators LasR and RhlR (Pessi and Haas, 2000), as well as AlgR (Carterson et al., 2004; Cody et al., 2009). The *hcnA* promoter contains three transcriptional start sites (T1–T3) (Pessi and Haas, 2000; Cody et al., 2009). Transcription from T1 is regulated by both LasR and RhlR, as this promoter region has corresponding luxα and luxβ binding sequences for these quorum-sensing regulators. Transcription from T2 is expressed microaerobically, and maximal transcription requires a combination of LasR, RhlR, and Anr proteins (Pessi and Haas, 2000).

In *P. aeruginosa* strain PAO1, T3 is located upstream of the luxβ box and is controlled by AlgR. The *hcnA*-ABS (5′-CCGTCGTTC-3′) is located −160 bp upstream of T3 (Figure 3A) (Carterson et al., 2004; Cody et al., 2009). This AlgR binding sequence is located immediately downstream of PA2192 (encoding a hypothetal protein), and *exoY* (a secreted adenylate cyclase). Interestingly, AlgR regulation of the *hcnA* gene is strain-dependent. Several *P. aeruginosa* strains contain chromosomal deletions of a region that would otherwise encode an AlgR binding site within the *hcnA* promoter region. Some strains (i.e., PA14, PAK) as well as several CF isolates have regions flanking *exoY* deleted (in the ORFs of PA2192 and PA2190) (Wolfgang et al., 2003a), which remove the AlgR-dependent *hcnA*-ABS from the chromosome. As a consequence, cyanide production in strains with such deletions are responsive to Anr and the quorum sensing regulators, but not responsive to AlgR (Cody et al., 2009).

Mucoid *P. aeruginosa* express and produce higher levels of *hcnA* and HCN as compared to isogenic non-mucoid strains (Firoved and Deretic, 2003; Carterson et al., 2004). AlgR positively regulates *hcnA* transcription in mucoid strains; inactivation of the *algR* gene decreases *hcnA* mRNA and HCN production (Carterson et al., 2004). In contrast, deletion of *algR* in non-mucoid strains resulted in increased HCN production, indicating that AlgR can represses *hcnA* in non-mucoid backgrounds (Cody et al., 2009). Additionally, AlgR phosphorylation is required for this repression, as non-mucoid strains expressing *algR*D54N displays increased HCN production. The reason for the apparent switch in phenotypes between mucoid and non-mucoid cells is currently unknown, but it may be due to effects of the other regulators that control the *hcnA* promoter (Cody et al., 2009). A possible system that could mediate this differential expression of *hcnA* is the RsmAYZ system, as RsmA has been demonstrated to post-translationally affect the expression of *hcnA* (Pessi and Haas, 2001; Pessi et al., 2001). As described below, AlgR regulation of the type III secretion system (T3SS) is likewise mediated through the RsmA/Y/Z system.

## AlgR and the type III secretion system

The type III secretion system (T3SS) is a needle-like structure that injects effector proteins (e.g., toxins) into host cells. Bacterial surface-contact, and low calcium concentrations in laboratory settings induce the expression of the T3SS in non-mucoid *P. aeruginosa* (Iglewski et al., 1978; Vallis et al., 1999). Expression of some acute virulence factors, including the T3SS machinery, is inversely correlated with the mucoid status of the organism (Mohr et al., 1990b; Wu et al., 2004). The reason for the inverse correlation between acute virulence factors and mucoidy could be that down regulation of the expression of virulence factors helps the organism to evade the host immune response, and to concentrate its energy into alginate production.

AlgR plays an important role in suppressing the T3SS in the context of a mucoid (*mucA*) background (Wu et al., 2004; Yahr and Wolfgang, 2006; Jones et al., 2010; Intile et al., 2014). Using the main transcriptional regulator of the T3SS (encoded by *exsA*) and expression of T3SS effector protein ExoS as readouts, *P. aeruginosa* carrying a *mucA22* allele (conferring mucoidy) exhibits suppressed T3SS expression as compared to non-mucoid organisms. Meanwhile, a *mucA22ΔalgR* double-deletion restored both *exsA* and *exoS* expression to wild type (in strain PAK) levels, and plasmid-expression of *algR* reduced *exsA* and *exoS* expression, indicating that AlgR represses these T3SS genes (Wu et al., 2004).

As discussed previously, the AlgU regulon is highly active in mucoid cells, leading to alginate biosynthesis and increased *algR* transcription (Mohr et al., 1990a, 1991, 1992; Martin et al., 1993b, 1994). However, AlgR-dependent suppression of the T3SS is unrelated to alginate production, as a *mucA22ΔalgD* allele has no affect on T3SS expression. These data indicate that AlgR is involved with T3SS modulation under conditions in which AlgU is activated (Wu et al., 2004).

Further studies showed that AlgR modulates T3SS expression indirectly through two mechanisms. The *P. aeruginosa* cAMP-responsive protein called virulence factor regulator (Vfr) is a cAMP-binding transcriptional regulator (West et al., 1994). Expression of *cyaA* or *cyaB* encoding adenylate cyclases increases intracellular cAMP levels, leading to higher Vfr activity, which results in activation of the T3SS (Wolfgang et al., 2003b). AlgR regulates the T3SS through control of Vfr. Deletion of both *algZ* and *algR* (a Δ*algZΔalgR* strain) increased transcriptional activity from a Vfr-responsive promoter, while *algZR* expression from a plasmid decreased transcriptional activity from this promoter (Jones et al., 2010). These results indicate that AlgZR repression of the T3SS could be mediated through its repression of Vfr.

AlgR is also proposed to regulate the T3SS through the RsmAYZ small RNA system (composed of the regulatory protein RsmA, and the small RNAs RsmY and RsmZ) (Jones et al., 2010; Intile et al., 2014). RsmA is an RNA-binding translational regulator that normally represses translation of its target transcripts. The small RNAs encoded by *rsmY* and *rsmZ* typically inhibit RsmA activity by competitively binding to the protein, thereby repressing RsmA activity (and de-repressing translation). RsmA (CsrA) proteins can also activate gene expression by altering mRNA secondary structure, mRNA stability, and/or ribosomal recruitment (Wei et al., 2001; Patterson-Fortin et al., 2013).

It is suspected that AlgR indirectly represses the T3SS by regulating *rsmA, rsmY*, and *rsmZ* expression. The evidence for this pathway is that *rsmA, rsmY*, and *rsmZ* transcription increased when *algR* is over-expressed, and that expression of AlgR in the mucoid background is correlated with increased expression of *rsmA, rsmY*, and *rsmZ* (Jones et al., 2010; Intile et al., 2014). RsmA-regulated transcripts are usually repressed by free-RsmA (e.g., *tssA1*, encoding a type VI secretion protein). However, recent work showed that *exsCEBA* expression (encoding regulatory proteins for T3SS activation) was activated either directly or indirectly by free-RsmA. The authors propose that small changes in RsmA availability could explain the target gene's different responses to RsmA/RsmY/RsmZ (Jones et al., 2010; Intile et al., 2014). In other words, AlgR may activate RsmA, RsmY, and RsmZ (and because all three are expressed, RsmA is competitively bound and inhibited by RsmY and RsmZ leading to relatively decreased levels of free-RsmA and non-activation of the T3SS in the mucoid background (Jones et al., 2010; Intile et al., 2014). Altogether, AlgR represses the T3SS in the mucoid background through Vfr and RsmA/Y/Z. More work is required to determine whether AlgR directly regulates Vfr or RsmA/Y/Z, or if there is an indirect component to this regulation that ultimately results in repression of the T3SS in the mucoid background.

AlgR's repressive role on T3SS is in contrast to its activator role on alginate production. In addition, AlgR has dual functions as a repressor and activator of HCN production in non-mucoid and mucoid backgrounds, respectively. In light of recent evidence of AlgR's effect on T3SS through the RsmA/Y/Z system, and the fact that RsmA also regulates *hcnA* expression, we speculate that AlgR may play a key role in influencing the expression of other virulence factors such as HCN production through the RsmA/Y/Z system.

## AlgR controls the expression of many virulence determinants

**Figure 5:**
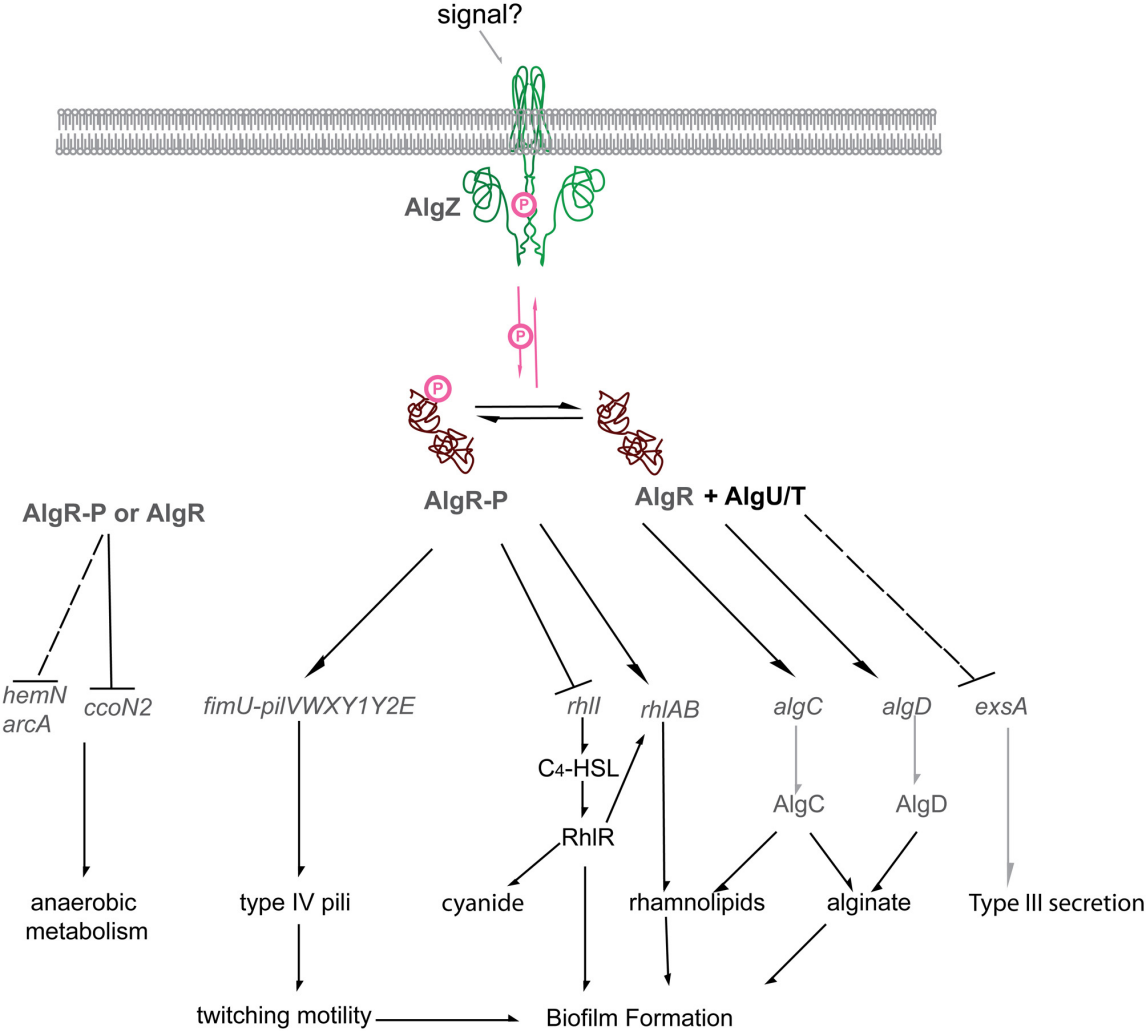
**The *P. aeruginosa AlgR regulon***. Diagrammatic representation of the current AlgR regulon. Phosphorylated AlgR activates expression of the pseudopilin operon *fimU pilVWXY1Y2*, as well as modulates the expression of *rhlA*, but represses *rhlI*. Unphosphorylated AlgR activates expression of alginate biosynthetic genes *algD* and *algC* but represses the expression of the type III secretion system through RsmA/RsmY/RsmZ. Arrows, AlgR stimulated gene expression; Block arrows, AlgR repressed gene expression; AlgR-P, phosphorylated AlgR; AlgR, unphosphorylated AlgR; AlgR + AlgU/T, unphosphorylated AlgR and the alternative sigma factor AlgU (AlgT).

AlgR globally affects transcription of a broad range of virulence factors including alginate production, twitching motility, the Rhl quorum sensing system, rhamnolipid production, T3SS, biofilm formation and hydrogen cyanide production (Figure 5). Therefore, it was ascertained whether or not *algR* is required for virulence. A component of *P. aeruginosa* virulence is its ability to survive reactive oxygen intermediates. AlgR represses genes required for survival against oxidative stress (Lizewski et al., 2002). Strain PAO700 (*algR::Gm*) was more susceptible to hypochlorite treatment, but more resistant to both hydrogen peroxide and myeloperoxidase compared to its isogenic PAO1 parental strain. The *algR* mutant was more resistant to killing by murine macrophages and primary human neutrophils, indicating that AlgR may normally repress the genes that are necessary to survive exposure to primary host response cells (Lizewski et al., 2002). On a related note, it was also demonstrated that a transposon insertion in *algR* (in strain PA14) results in increased persistence (persistence in this study was defined by the ability to grow in 5 ug/mL of oflaxicin) (De Groote et al., 2009). Although the direct mechanism is unknown, it supports previous data indicating AlgR may also regulate the expression of antibiotic resistance genes (Lizewski et al., 2002).

The *algR* gene was required for virulence in two different murine infection models and a competition assay. Strain PAO700 (*algR::Gm*) was tested in acute septicemia and acute pneumonia models of infection in C57BL/6j mice. Mice infected by intraperitoneal injection with PAO700 show increased survival as compared to mice infected with the wild type PAO1 strain. The same *algR* inactivated strain was cleared more quickly from murine lungs after acute infection. Co-infection with PAO700 and PAO1 resulted in decreased recovery of PAO700 as compared to wild type from the mice, suggesting an *algR* requirement for maximal virulence in these models. Interestingly, constitutive expression of *algR* decreased virulence as well suggesting that AlgR protein levels in *P. aeruginosa* need to be balanced for pathogenicity of the organism (Lizewski et al., 2002). The repression of virulence during increased AlgR expression is consistent with its regulation of the alginate system, as increased AlgR expression suppressed alginate production (Deretic and Konyecsni, 1989).

In all, AlgR appears to be a global regulator of *P. aeruginosa* virulence, and likely affects the organism's fitness in a broad manner. AlgR is also required for alginate production in a mucoid background, suggesting its importance in chronic pulmonary infections of CF victims. This response regulator is required for two phenotypes: twitching motility via *fimU* and alginate production through *algD.* However, global transcriptome analysis indicated that it controls many different genes, including those associated with quorum sensing, type IV pili, type III secretion system, anaerobic metabolism, cyanide and rhamnolipid production (Figure 5). Several questions remain: Does AlgR control these different *P. aeruginosa* genes directly or indirectly? What is/are the signal(s) to which AlgZ and ultimately AlgR respond? How is AlgR able to activate transcription regardless of its phosphorylation state? Current studies are underway to answer these questions.

### Conflict of interest statement

The authors declare that the research was conducted in the absence of any commercial or financial relationships that could be construed as a potential conflict of interest.
